# Comparison of loop-mediated isothermal amplification and conventional PCR tests for diagnosis of common *Brucella* species

**DOI:** 10.1186/s13104-020-05377-8

**Published:** 2020-11-13

**Authors:** Ali Moeini-Zanjani, Abazar Pournajaf, Elaheh Ferdosi-Shahandashti, Mehrdad Gholami, Faramarz Masjedian, Soraya Khafri, Ramazan Rajabnia

**Affiliations:** 1 Department of Microbiology, Faculty of Medicine, Babol University of Medical Sciences, Babol, Iran; 2 Infectious Diseases and Tropical Medicine Research Center, Babol University of Medical Sciences, Babol, Iran; 3 Department of Medical Biotechnology, Faculty of Medicine, Babol University of Medical Sciences, Babol, Iran; 4 Department of Microbiology and Virology, Faculty of Medicine, Mazandaran University of Medical Sciences, Sari, Iran; 5 Department of Microbiology, Faculty of Medicine, Iran University of Medical Sciences, Tehran, Iran; 6 Department of Biostatistics and Epidemiology, Faculty of Medicine, Babol University of Medical Sciences, Babol, Iran

**Keywords:** Brucellosis, Diagnosis, PCR, LAMP

## Abstract

**Objective:**

Rapid, reliable, and affordable detection of *Brucella* species via the molecular methods remains a challenge. In recent years, loop-mediated isothermal amplification (LAMP) is a functional nucleic acid amplification technique offering a substitute to polymerase chain reaction (PCR). So, we compared the LAMP assay with the conventional PCR for the identification of common *Brucella* species in Iran. In this study, LAMP assay was comprehensively evaluated against the common PCR method. A group of specific LAMP primers were used to amplify a highly specific fragment from the sequence of the *Brucella abortus, bcsp31* gene. Sensitivity and specificity values of tests were done with a set of 78 (50 *Brucella* and 28 non-*Brucella*) strains.

**Results:**

A dilution series of *B. abortus* DNA indicated that the LAMP reaction could reliably detect 10 (fg/µl) DNA target copies per reaction within 36 min, which is 10 times greater than the PCR assay. In summary, we conclude that LAMP assay provide accurate and fast test results to identify of common *Brucella* species in low-complexity labs, mainly in low and lower middle income countries.

## Introduction

*Brucella* species are small, coccobacilli, Gram-negative, absolute aerobic and non-moving bacteria which causes undulant fever in humans and leads to abortion and infertility in animals [1]. *Brucella* can be transmitted to humans through direct contact with animals or their products that are contaminated with these bacteria [2]. *Brucella* genus has six species that cannot be distinguished from each other due to the close phenotypic and antigenic similarities with conventional microbiological methods [3, 4]. From these six species, [*B. abortus*, *B. melitensis*, *B. suis* and *B. canis*] generally causes human infection [5]. About half a million cases of human brucellosis are reported annually which is estimated to be 10–25% less than the real number of the 1997 world health organization (WHO) report [6]. The centers for disease control and prevention strategic planning group as listed *B. abortus*, *B. melitensis*, *B. suis* as category B biothreat agents [5, 7]. There are currently three main methods for the identification of brucellosis [8]. Overall, in human brucellosis, isolation of the bacterium by blood culture is considered as the gold standard for laboratory diagnosis, but the procedure is time consuming [9, 10], and has a low sensitivity in the range of 15–70% depending on the bacterial species and infectious phase [11]. On the other hand, this method, due to class III biosafety of *Brucella* is very dangerous and pollutant for laboratory personnel [12, 13]. Serological examinations are relatively fast to accomplish; however, their results are not satisfactory. One such example is that they have low sensitivity, due to the structural similarity of *Brucella* lipopolysaccharide (LPS) with other Gram-negative bacteria [3, 14]. Nucleic acid amplification tests (NAATs), such as the polymerase chain reaction (PCR) with high sensitivity and specificity have been developed to detect *Brucella* DNA in human specimens [15, 16, 17]. PCR and other molecular techniques have their own disadvantages; for example; most molecular techniques require a thermal cycler machine, which is not feasible for laboratories in deprived areas such as rural laboratories [18]. The LAMP system has been developed as a new diagnostic technique which could replicate the target DNA without the need for a thermal cycler. In this method with unique Bst DNA polymerase large fragment characteristics such as autoclaving and strand displacement, it became possible to eliminate the thermal cycler and replaced it with thermal block or bain-marie, it was also possible to see the results by visual inspection. because of turbidity generated in positive samples [19, 20]. This technique has already been evaluated and tested to identify and detect different bacteria and viruses and its high sensitivity and specificity have been proven [21–24]. So, we compared the LAMP assay with the conventional PCR method for the identification of common *Brucella* species in Iran.

## Main text

### Methods

#### Bacterial strains and sample processing

In this study, to standardize the LAMP protocols, 78 bacterial strains including 50 *Brucella* and 28 non-*Brucella* strains were analyzed (details mentioned in Table 1). *Brucella* strains was cultured in a BSL2 laboratory on 5% sheep blood agar medium (Merck, Germany) and on *Brucella* agar medium with 5% sheep blood (Merck, Germany) under 5% CO_2_ in an-anaerobic jar for 36 h at 37 ºC. Other non-*Brucella* strains were cultured in trypticase soy agar (Merck, Germany) and deleted replasing supplemented with 5% sheep blood agar for 18 h at 37 ºC.

**Table 1:** Bacterial strains used in this study and the results of PCR and LAMP amplification

Strains	Species (biovar)	No. of strains	Source	LAMP results	PCR results
*B. abortus*	1	1	S99 (Reference)	+	+
*B. abortus*	1	1	S19 (vaccine strain)	+	+
*B. abortus*	2	1	Clinical isolate	+	+
*B. abortus*	3	18	Clinical isolate	+	+
*B. abortus*	3	7	Animal isolate	+	+
*B. melitensis*	1	1	16 M (ATCC23456)	+	+
*B. melitensis*	1	13	Clinical isolate	+	+
*B. melitensis*	1	8	Animal isolate	+	+
*Escherichia coli*	O157:H7	4	Clinical isolate	−	−
*Staphylococcus aureus*		4	Clinical isolate	−	−
*Vibrio cholerae*	O1	4	Clinical isolate	−	−
*Klebsiella pneumoniae*		4	Clinical isolate	−	−
*Acinetobacter baumannii*		4	Clinical isolate	−	−
*Pseudomonas*		4	Clinical isolate	−	−
*Shigella flexineri*		4	Clinical isolate	−	−

#### DNA extraction procedures

Briefly, a loopful of colonies were aseptically collected from the plates and suspended in 5 mL pf phosphate buffered saline (PBS) until its opacity reaches #2 McFarland standard turbidity. After vortexing, genomic DNA of strains were extracted with the High Pure PCR Template Preparation Kit (Roche, Germany), according to the manufacturer’s instructions. The absorbance ratio (A260/280) is used to evaluate the purity of DNA.

#### Primers

We tried to detect the same gene in each two techniques for minimization of the variables, so the *bcsp31* gene selected for the detection in both techniques. The primers sequences used for LAMP and PCR was shown in Table2.

**Table 2:** Sequences of primers used for LAMP and PCR assay

Assay	Primer	Sequence	Amplicon size (bp)	Reference
LAMP	F3	5′‐GCTTTACGCAGTCAGACGT‐3′	189	[25]
	B3	5′‐GCTCATCCAGCGAAACGC‐3′		
	FIP	5′‐AGGCGCAAATCTTCCACCTTGCGCCTATTGGGCCTATAACGG‐3′		
	BIP	5′‐GGCGACGCTTTACCCGGAAATTCAGGTCTGCGACCGAT‐3′		
	LF	5′‐CCTTGCCATCATAAAGGCC‐3′		
	LB	5′‐CGTAAGGATGCAAACATCAA‐3′		
PCR	B4	5′-TGGCTCGGTTGCCAATATCAA-3′	223	[15]
	B5	5′-CGCGCTTGCCTTTCAGGTCTG-3′		

#### Conventional PCR

The PCR assay was carried out as previously described by Baily et al. [15]. Amplification targeting *bcsp31* gene was performed in a Techne TC-512 thermal cycler (Eppendorf, Hamburg, Germany) according to the conditions mentioned in Table 3.

**Table 3:** PCR condition for *bcsp31* template

Feature	Temperature (°C)	Time
Gene (*bcsp31*)		
Initial denaturation	95	5 min
Denaturation	95	60 s
Annealing	65	30 s
Extension	72	60 s
Final extension	72	6 min
Cycle	35	–

#### LAMP reaction optimization

We used six primers for LAMP assay in this study, LAMP outer primers (F3 and B3), forward inner primer (FIP) and backward inner primer (BIP), which identify four different fragments on the DNA target sequence, and two loop primers (LF and LB) to increase proliferation speed. Also, modifying concentration of reaction components and conditions such as reaction time (20–50 min), amplification temperatures (61–67 °C), concentration of dNTPs (0 to 2 mM), MgSO4 (0–6.4 mM) and Bst polymerase (2–12 Unit) were chosen to assist in optimizing LAMP procedure in targeting the *bcsp31* gene. The optimized reaction mixture contained 5 pmol l^−1^ each of outer primers (F3 and B3), 40 pmol l^−1^ each of inner primers (FIP and BIP), 20 pmol l^−1^ each of loop primers (LF and LB), 1.4 mmol l^−1^ each deoxynucleoside triphosphates, 0.8 mol l^−1^betain (Sigma, B0300,St. Louis, USA), 20 mmol l^−1^Tris–HCl, 10 mmol l^−1^ (NH4)2SO4, 10 mmol l^−1^KCl, 8 mmol l^−1^ MgSO4, 0.1% Triton X-100, 8 units of Bst polymerase (New EnglandBiolabs, M0275S, Beverly, USA) and 2 µl of genomic DNA. Before adding the Bst DNA polymerase, reaction mixture was heated up at 95 °C for 5 min in a Thermoblock heat system, and then cooled on ice for 10 min to provide an appropriate environment for adding Bst. Subsequently, the mixture was incubated at 63 ºC for 35 min and then, tubes heated at 95 °C for 2 min to stop the reaction. Five microliters of the product were subjected to 2% agarose gel electrophoresis.

#### Results

All the 50 strains of 4 serotypes of *Brucella* were shown to be positive by LAMP.

#### Conventional PCR

The PCR was performed on 78 species, and a 223 bp bands was observed in all of *Brucella spp*, on 1.5% agarose gel. Whereas, no band was seen in other species. The sensitivity and specificity of the primers used in this study have been evaluated by Baily et al. [15]. The detection limit for the PCR assay in the mentioned conditions was 100 fg.

#### Establishment of *BCSP31* LAMP and product confirmation

In this research, we tried our best to minimize the *Brucella* DNA amplification time in the LAMP assay. So, in deprived areas of Iran, laboratories can measure human or animal contamination in the shortest time with the protocol described in this study. By changing the LAMP assay reaction [termination step protocol from 80 °C in 5 min to 95 °C in 2 min] we obtained similar results. Thus we replaced the new modified protocol with the general protocol. To set up and optimization, we used *B. melitensis* 16 M (ATCC 23456) DNA as template. The LAMP assay successfully amplified the target (*bcsp31* gene) at 63ºC in 35 min. The well-defined bands patterns, which is a trait of the LAMP reaction and shows the forming DNA stem–loop with inverted repeats of the target sequence [19] was checked using gel electrophoresis method.

#### Sensitivity of LAMP reaction

After adjusting the best conditions for the LAMP reaction, the lower detection limit of the LAMP was measured using tenfold serial dilution (initial concentration of 10 ng) of the DNA samples for *bcsp31* gene. Ladder-like pattern on 2% agarose gel was identified by target DNA of as low as 10 fg. Also, sensitivity of minimal detectable rate of LAMP test in compare with PCR is shown in Additional file 1: Figure S1. As shown in the figure, the minimal detectable rate of the LAMP test is 10 fg/µl. Therefore, it can be concluded that the minimal detectable rate of LAMP test is 10-times higher than PCR and can be detect a less amount of bacterial genome in the sample. So, LAMP test is more sensitive than PCR.

#### Naked-eye detection of positive *bcsp31* LAMP amplification

Under situations where detection and urgent care requires rapid response, it is greatly favorable for specify all positive samples as soon as possible. This is one of the advantages of the LAMP assay versus PCR. To achieve this important, the products inside the 0.2 mL microtubes were placed under white light and the turbidity caused by magnesium pyrophosphate (Mg_2_P_2_O_7_) in positive samples was observed with the naked eye. Products were also observed by adding 1 to 100 diluted fluorescent detection reagent (SYBR Green I, S9430, Sigma-Aldrich, Germany) under normal, and UV light. There was no difference between the LAMP results detected by fluorescence and turbidity.

#### Assay specificity through several types of bacterial spp

To assess the specificity of the LAMP reaction, all 78 bacterial strains were tested by *bcsp31*gene-based LAMP assay. The results of this assay are shown in Table 1; the test result for non-*Brucella* species was negative. Our results showed that the P-1 primer set successfully and specifically amplified *bcsp31* gene of *Brucella* species in vaccine strains, clinical and animal isolates. Whereas other non-*Brucella* strains did not show any turbidity, fluorescent and any bands on the agarose gel electrophoresis under equal conditions.

### Discussion

This is the primary description on the employment of LAMP to the identification of common species of *Brucella* based on the *bcsp31* gene in Iran. Brucellosis remains a neglected illness in the developing countries. Due to the high prevalence of Brucellosis early diagnosis in conjugation with timely medical intervention is necessary to prevention and control of the infectious disease [26]. The Rose Bengal test (RBT) as a easy method for the identification of specific antibodies against *Brucella*. However, their fruitfulness is restricted via elevated frequencies of *Brucella*‐specific antibody titer in high-brucellosis burden countries and it low sensitivity at acute phase of disease [27, 28]. Furthermore, the growth of *Brucella* *spp*. is time consuming and complex, subsequently bacteriological culture and microscopic examination are cumbersome and difficult [29]. Our major purpose of this work was to assess and compare the diagnostic capabilities of two different molecular detection methods, i.e. LAMP and PCR techniques and to find out whether the LAMP technique is a good alternative to the PCR. We used direct culture test as the “Gold standard” for the preparation of fresh DNA and the uniformization of the terms of the techniques. The LAMP assay is advantageous in compare to the PCR, because of its feasible, easy construction, quick answer and visual recognition. Its simplicity and using low-cost equipment, including laboratory water bath that prepare a stable heat of 63 °C is acceptable for the test. Compared to the PCR, the LAMP results is straightly observable with the unaided eye negating the necessity for electrophoretic investigation [19]. Moreover, the LAMP assay unlike other molecular techniques does not require special and expensive devices such as thermal cycler and can be performed in low-budget labs in deprived areas, which there is a probability of an outbreak or expose to brucellosis. Characteristics evaluated for the comparison of two techniques in order to overcome one over another in the studies includes the duration of the amplification, the sensitivity, specificity of the technique and the limit of detection. The PCR technique lasted approximately 90 min and 100 fg of *Brucella* DNA was successfully completed. In addition, non-specific responses to multiple negative controls were not observed. In the LAMP assay, 10 fg of *Brucella* DNA was favorably amplified during 35 min and, the primers used did not have any non-specific reaction to bacterial DNA that was used as negative controls. In comparison of these two assays, the PCR took about 90 min, while the *Brucella* LAMP can be finished within 35 min also according to the limit of detection in two assays the sensitivity of the LAMP assay was 10 times higher to that of PCR. About the specificity of the two techniques, each of the two techniques does not have any nonspecific reaction with multiple negative controls and their specificity was almost equal. The *Brucella* LAMP technique examined in the work is a quick, highly specific and sensitive method that can be substituted for PCR assays in the low-budget labs of Iran and are able to identify native strains of *Brucella* in Iran. This is a suitable system for peripheral laboratories to diagnosis and investigation of human brucellosis in endemic setting.

## Limitations

The assay should be evaluated using blood or other clinical specimens from infected patients.

## Supplementary information

**Additional file 1: Figure S1.** Sensitivity of minimal detectable rate of LAMP test in compare with PCR. (A); the minimal detectable rate of Brucella DNA in PCR technique is 100 fg / μl, (B); detection of 10 fg / μl bacteria genome in LAMP test. M; 100 bp DNA size marker (SinaClon Bioscience Co, Iran), C- (negative control).

## Data Availability

All data generated or analyzed during this study are included in this published article.

## Abbreviations

LAMP: Loop-mediated isothermal amplification
PCR: Polymerase chain reaction
WHO: World health organization
PBS: Phosphate buffered saline
LPS: Lipopolysaccharide
RBT: Rose Bengal test
