# Supplementary material for: Comparison of loop-mediated isothermal amplification and conventional PCR tests for diagnosis of common Brucella species
Source: BMC Res Notes. 2020 Nov 13;13:533. doi: 10.1186/s13104-020-05377-8 (PMC7666441; doi:10.1186/s13104-020-05377-8)
Supplement: Supplementary file 1 — Additional file 1: Figure S1. Sensitivity of minimal detectable rate of LAMP test in compare with PCR. (A); the minimal detectable rate of Brucella DNA in PCR technique is 100 fg / μl, (B); detection of 10 fg / μl bacteria genome in LAMP test. M; 100 bp DNA size marker (SinaClon Bioscience Co, Iran), C- (negative control). [file 13104_2020_5377_MOESM1_ESM.pdf]

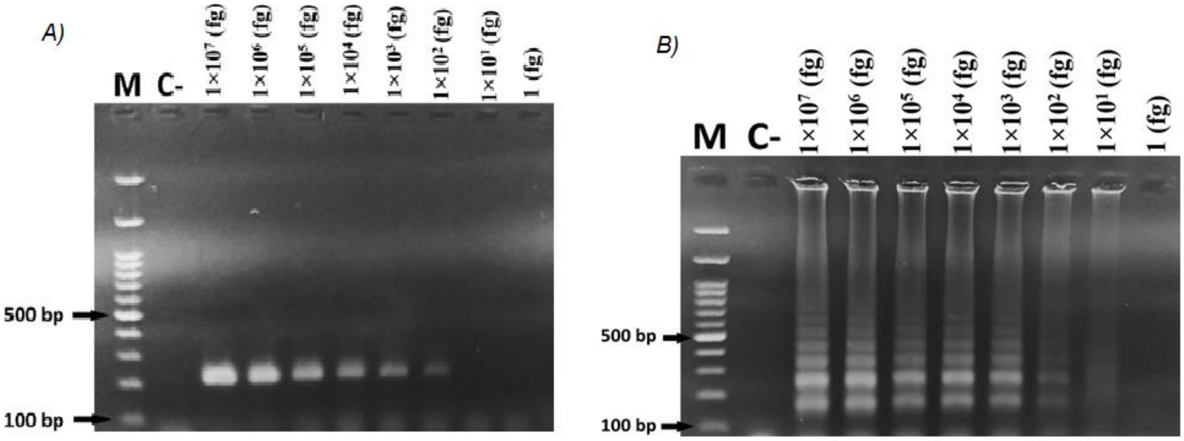

FigureS1. Sensitivity of minimal detectable rate of LAMP test in compare with PCR. (A); the minimal detectable rate of Brucella DNA in PCR technique is 100 fg / µl, (B); detection of 10 fg / µl bacteria genome in LAMP test. M; 100 bp DNA size marker (SinaClon Bioscience Co, Iran), C- (negative control).
